# A Critical Analysis of the COVID-19 Hospitalization Network in Countries with Limited Resources

**DOI:** 10.3390/ijerph19073872

**Published:** 2022-03-24

**Authors:** Marcio L. V. Araujo, José G. V. Miranda, Rodrigo N. Vasconcelos, Elaine C. B. Cambui, Raphael S. Rosário, Márcio C. F. Macedo, Antonio C. Bandeira, Márcia S. P. L. Souza, Ana C. F. N. Silva, Aloisio S. Nascimento Filho, Thiago B. Murari, Eduardo M. F. Jorge, Hugo Saba

**Affiliations:** 1 Modelagem Computacional e Tecnologia Industrial (PPG MCTI), Centro Universitário SENAI CIMATEC, Av. Orlando Gomes, 1845—Piatã, Salvador 41650-010, Brazil; hugosaba@gmail.com; 2 Department of Computer Science, Federal Institute of Bahia, Rua São Cristóvão, s/n—Novo Horizonte, Lauro de Freitas 42700-000, Brazil; 3 Núcleo de Pesquisa Aplicada e Inovação (NPAI), Universidade do Estado da Bahia—UNEB/Centro Universitário SENAI CIMATEC, Salvador 41650-010, Brazil; aloisio.nascimento@gmail.com (A.S.N.F.); mura.learning@gmail.com (T.B.M.); emjorge1974@gmail.com (E.M.F.J.); 4 Department of Physics, Federal University of Bahia, Campus Universitário de Ondina—Ondina, Salvador 40210-340, Brazil; vivasm@gmail.com (J.G.V.M.); phaoso@gmail.com (R.S.R.); marciocfmacedo@gmail.com (M.C.F.M.); 5 Department of Earth and Environmental Sciences, State University of Feira de Santana, Av. Transnordestina, s/n—Feira de Santana, Novo Horizonte 44036-900, Brazil; rnvuefsppgm@gmail.com; 6 Department of Ecology, Federal University of Bahia, Campus Universitário de Ondina—Ondina, Salvador 40210-340, Brazil; elainecambui@gmail.com; 7 Health Secretary of the State of Bahia, 4ª Avenida Centro Administrativo da Bahia, 400—Centro Administrativo da Bahia, Salvador 40301-110, Brazil; antoniobandeira@gmail.com (A.C.B.); marcia.saopedro@saude.ba.gov.br (M.S.P.L.S.); anaclaudianunes@hotmail.com (A.C.F.N.S.); 8 Gestão e Tecnologia Industrial (PPG GETEC), Centro Universitário SENAI CIMATEC, Av. Orlando Gomes, 1845—Piatã, Salvador 41650-010, Brazil; 9 Department of Exact and Earth Sciences, University of the State of Bahia, R. Silveira Martins, 2555—Cabula, Salvador 41180-045, Brazil

**Keywords:** government, hospitalization, pandemics, public policy, transportation

## Abstract

To effectively combat the COVID-19 pandemic, countries with limited resources could only allocate intensive and non-intensive care units to a low number of regions. In this work, we evaluated the actual displacement of infected patients in search of care, aiming to understand how the networks of planned and actual hospitalizations take place. To assess the flow of hospitalizations outside the place of residence, we used the concepts of complex networks. Our findings indicate that the current distribution of health facilities in Bahia, Brazil, is not sufficient to effectively reduce the distances traveled by patients with COVID-19 who require hospitalization. We believe that unnecessary trips to distant hospitals can put both the sick and the healthy involved in the transport process at risk, further delaying the stabilization of the COVID-19 pandemic in each region of the state of Bahia. From the results found, we concluded that, to mitigate this situation, the implementation of health units in countries with limited resources should be based on scientific methods, and international collaborations should be established.

## 1. Introduction

The state of Bahia, Brazil, has a total area of approximately 565,000 square kilometers and, by the end of 2020, had an estimated population of about 15 million people, which is superior to the estimated population of European countries, such as Belgium, Greece, Sweden, and Portugal [1]. The human development index (HDI) of the state of Bahia is 0.660, similar to other countries with limited resources such as Guatemala, Honduras, India, Bangladesh, and Morocco. In this sense, while in normal conditions it is already difficult to provide free, high-quality health care services to the population that live in Bahia, on 11 March 2020, with the World Health Organization (WHO) announcement of the coronavirus disease 2019 (COVID-19) outbreak, caused by the new severe acute respiratory syndrome coronavirus 2 (SARS-CoV-2), as a pandemic, the provision of high-quality health care services has become even more challenging, since the high transmissibility of SARS-CoV-2 may quickly overload the health care services of a state or a country [2].

Possibly inspired by the National Health Service of the United Kingdom [3], the Brazilian National Health System (Sistema Único de Saúde—SUS) is a public health system created by the Constitution of the Federative Republic of Brazil [4]. The SUS is a universal access system, which allows any Brazilian to use low-, medium-, and high-complexity health services. In addition, management is divided between federal, state, and municipal sectors. States and municipalities have autonomy in defining policies in favor of health [5]. Previous studies in Brazil have found that, although there was a favorable expansion in Universal Health Coverage (UHC), structural problems remained in the SUS, from gaps in organization and governance to a scarcity of public resources. Thus, it is evident that there are regional disparities in terms of health services [6]. However, the geographic and economic differences do not allow for a comparison between these countries in terms of the flow of hospitalization. Previous works deal with the use of resources in health services [7,8]. This work aims to analyze the flow of hospitalizations in a country with limited resources. The displacement of patients receiving hospital treatment during the COVID-19 pandemic has proved to be a problem for some countries [9,10].

In the state of Bahia, Brazil, the management of SUS is done by the state government of Bahia, which must provide financial resources and stimulate the municipalities of Bahia to achieve the responsible management of their health care services, and must also assume that responsibility in the case some of those municipalities are not able to achieve that goal [5]. As resources are limited and the territorial space of Bahia is large, the creation of health units, in a few areas, is presented as a single alternative, so large displacements of infected patients are inevitable.

By the end of October 2020, following the State Contingency Plan for Confrontation of the New Coronavirus SARS-CoV-2 [11], the state government of Bahia distributed 2286 intensive and non-intensive care units in 61 out of the 417 municipalities of Bahia, in order to provide better assistance to the COVID-19 patients that live in Bahia [12], 41% higher than the previous healthcare network. In Bahia, the room availability follows a protocol based on clinical severity, risk potential, health problems or degree of suffering [13]. Many of these health rooms were created to serve COVID-19 patients, as part of a municipal, state, and federal effort. Therefore, COVID-19 patients that reside in municipalities not capable of providing appropriate treatment or hospitalization for such a disease must travel to another municipality to be better assisted by SUS. In order to reduce the distance and duration of those travels, the state government of Bahia has distributed health care units into municipalities located into nine regions (i.e., North, Northeast, North-Central, East-Central, East, West, Southwest, South, and Extreme-South) that geographically divide the state of Bahia, such that COVID-19 patients could be ideally treated or hospitalized in health care units available at the regions where they reside, promoting a region-based control of the COVID-19 pandemic inside the state of Bahia.

Related work has already shown that we need to analyze transportation networks between the municipalities and states of a country [14,15,16,17] to be able to study the dynamics of dissemination of infectious diseases, such as COVID-19 [18,19,20,21,22,23,24], as well as to evaluate how we can guarantee a safe transportation for COVID-19 patients to be hospitalized in the health care units [25,26,27] of Bahia.

In this paper, we aim to evaluate whether the distribution of intensive and non-intensive care units among 61 out of the 417 municipalities in Bahia is sufficient to assist COVID-19 patients inside each one of the nine distinct regions that divide the state of Bahia. On the basis of a dataset provided by the Health Secretary of the State of Bahia (Secretária de Saúde do Estado da Bahia—SESAB) and by the Brazilian Ministry of Health (Ministério da Saúde do Brasil), from open health systems data, we have built a hospitalization network for COVID-19 patients in Bahia, Brazil, based on the concepts of complex networks and using geoprocessing. We have also analyzed whether COVID-19 patients are indeed being hospitalized in health care units located in the same region where they live, and if that is not the case, we determined the relation of importation and exportation of hospitalized COVID-19 patients between distinct regions in Bahia. The specificity of the study and the absence of an index that could be used to compare the level of care and hospitalization provided by a given region led to the development of the Degree of External Search for Hospitalization (DESH) index, used to estimate the saturation level of the municipalities that offer in-hospital assistance for COVID-19 patients that come from other regions of the state of Bahia.

The network approach allowed us to evaluate the relationship between the external demand for COVID-19 care units and the supply of these units in the region. The DESH measure does not represent a new topological index of the network, but rather a simple evaluation of the external pressure for care units in the municipality relativized with respect to the total number of care units available in it. We believe that the discussions presented in this paper may be helpful to the state government of Bahia, which may improve its decision-making process to effectively control the COVID-19 pandemic in Bahia, and we also believe that this kind of study may be replicated for other states and countries around the world to verify whether the hospitalization networks previously estimated by governments match the real ones obtained in practice.

## 2. Materials and Methods

### 2.1. Data Collection

In this study, we considered the COVID-19 patients that were hospitalized in intensive or non-intensive care units provided by SUS in the state of Bahia, Brazil, between 1 March 2020 and 30 July 2020, and that have been reported in the hospital systems. To build the hospitalization networks, the data represent all hospitalizations of patients affected by COVID-19 (4387) who were removed from their city of residence to another city. The objective was to measure the displacement of these patients due to the unavailability of health units in their municipality of residence.

### 2.2. Hospitalization Network

On the basis of the graph theory, we represent the hospitalization networks for COVID-19 patients in Bahia as a directed graph, where each node is assigned to a municipality in Bahia, each node’s size is directly proportional to its DESH index to be presented in the next subsection, each directed edge represents a travel going from an origin (i.e., municipality of residence) to a destination (i.e., municipality of hospitalization), and each directed edge is weighted by the number of patients that traveled between the corresponding pair of origin–destination municipalities. Hence, the hospitalization networks provides a visual representation of the patients that needed to be hospitalized because of the severity of COVID-19, but could not be hospitalized in their municipality of residence due to the unavailability of a health care unit at that location and at that moment.

### 2.3. Simulation of the Expected Network

In order to evaluate whether the strategy of the state government of Bahia to distribute health care units among the nine regions in Bahia was successful, we simulated the expected hospitalization networks idealized by the state government. To do so, we redirected each edge of the observed hospitalization networks to connect the node representing the municipality of residence of the patient to another node representing the closest municipality with an available health care unit, such that each redirected edge could represent the expected path traveled by a patient when searching for hospitalization by COVID-19 in Bahia.

### 2.4. DESH (Degree of External Search for Hospitalization) Index

To favor a comparison between the level of assistance and hospitalization provided by each region of the state of Bahia, we have developed the DESH index. With such an index, we can measure the saturation level of the 61 municipalities able to hospitalize COVID-19 patients. While each one of those 61 municipalities must provide assistance to the internal demand for hospitalization, by providing support to the patients that live inside the corresponding municipality, the DESH index only takes into account the external demand for hospitalization. In other words, this index measures how much each municipality is involved in the importation of COVID-19 patients provided by other municipalities that could be located inside or outside of the corresponding region of the state of Bahia.

The DESH index of a municipality i, or node i, with at least one intensive or non-intensive care unit available can be described in terms of Equation (1):

(1) $$DESH_{i}=\frac{\sum_{j=1}^{N-1}w{}_{ij}}{\gamma_{i}}$$

where $w_{ij}$ is the weight of a directed edge that connects node i to node *j* (i.e., the number of patients that traveled from municipality *j* to municipality *i*), *i* is the total number of intensive and non-intensive care units available at that municipality *i*, *N* is the total number of municipalities being evaluated, and $\gamma_{i}$ represents the municipalities.

Each one of the nine regions of Bahia is represented by a specific color. Each node (red circle) of the directed graph represents a municipality of Bahia. Each node size is directly proportional to its DESH index. The weight of each directed edge (black line) is directly proportional to the number of patients that have been transported to the corresponding municipality for hospitalization.

## 3. Results

**Figure 1:**
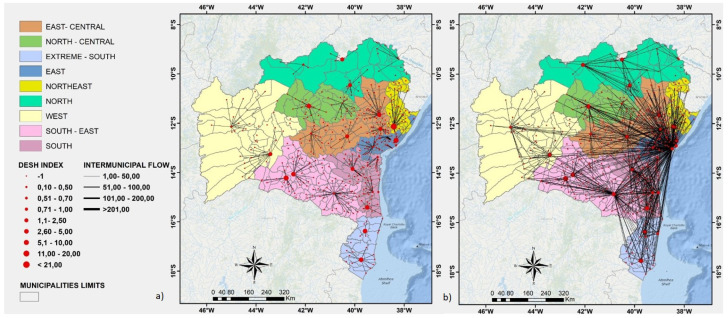
A visual comparison between the expected (**a**) and observed (**b**) hospitalization networks for COVID-19 patients in Bahia, Brazil. Source: Author.

As we can see in Figure 1a, according to the original planning of the state government of Bahia, even if not every one of the 417 municipalities of Bahia has a reference hospital or health care unit able to treat COVID-19 patients, the expected hospitalization networks for COVID-19 patients would be the one in which each patient would be hospitalized in the nearest reference unit available in the region where the patient is residing, such that COVID-19 patients would travel as minimum a distance as possible to be hospitalized, consequently allowing for a faster and more efficient treatment of those patients, as well as for a lower exposition to COVID-19 of the professionals involved in the transportation process. Hence, as can be seen by the weights of the directed edges shown in Figure 1a, once the health care units of each region would concentrate the hospitalization cases of the patients that live in the corresponding municipalities, the hospitalization networks would be more distributed all over the state, and the COVID-19 pandemic could be handled locally, per region of the state of Bahia. However, on the basis of the anonymized data collected from SESAB, we could estimate that the observed hospitalization networks for hospitalized patients are more similar to the one illustrated in Figure 1b. In this case, we can see that several COVID-19 patients need to travel from one region to another to be properly hospitalized, which suggests that some regions of the state of Bahia are not able to handle the high demands of hospitalization that may be happening due to COVID-19. In the ideal, expected scenario depicted in Figure 1a, each region would hospitalize only resident patients diagnosed with COVID-19. However, Table 1 shows that, while the North, East, and Southwest regions exported a few patients to the other regions of the state of Bahia, the North-Central, East-Central, and Northwest regions exported more than three times the number of patients that they could hospitalize. We also observed that both the East and Southwest regions concentrate the highest percentage of imported hospitalizations. In this case, it is worth noting that almost 50% of the hospitalizations done in the East region, which includes the capital of the state of Bahia, Salvador, are imported from other regions, while only 0.5% of the hospitalized cases are exported to other regions.

## 4. Discussion

**Figure 2:**
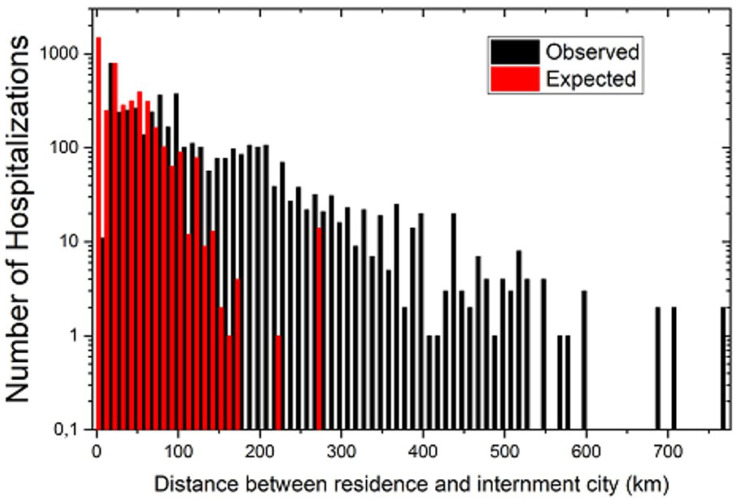
A histogram with the frequencies of expected (red) and observed (black) distances traveled by COVID-19 patients. Source: Author.

One of the main problems caused by such an unbalanced observed hospitalization networks is illustrated in Figure 2, which shows that, while in the expected hospitalization networks, some travel would be required to transport patients between the municipalities of the same region, in the observed scenario, more patients needed to travel longer distances to be hospitalized outside of their region of residence.

In our point of view, this unnecessary transportation of patients may affect the state of Bahia in two ways: First, this scenario may reduce the effectiveness in the reduction of the number of new cases of COVID-19 per region of the state of Bahia, since new patients diagnosed with COVID-19 might end up being hospitalized in another region that has already stabilized the COVID-19 pandemic, exposing the healthcare professionals of such a region to the coronavirus, which, once they are infected by COVID-19, could further disseminate the infectious disease to other people, contributing to a new rise in the number of new cases of COVID-19 per day. Second, this unnecessary transportation may result in additional costs for the state and the municipalities of Bahia, since they both are financially responsible for the management of the healthcare professionals and the infrastructure used to transport the patients to be hospitalized, and for the maintenance of the intensive and non-intensive care units that otherwise would be empty or at least less occupied, assuming a scenario in which a region is exporting new COVID-19 patients to be hospitalized to another region that has achieved stabilization with few new cases of COVID-19.

**Figure 3:**
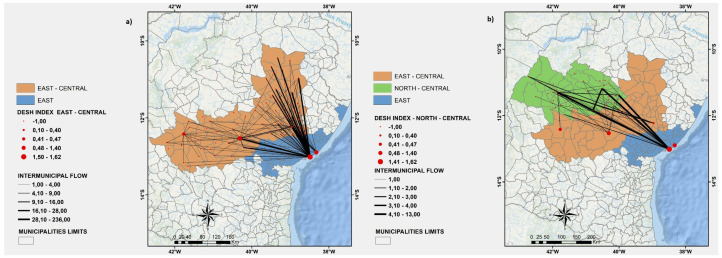
(**a**) A visualization of the East and East-Central regions. (**b**) A visualization of the East, East-Central, and North-Central regions. Source: Author.

**Figure 4:**
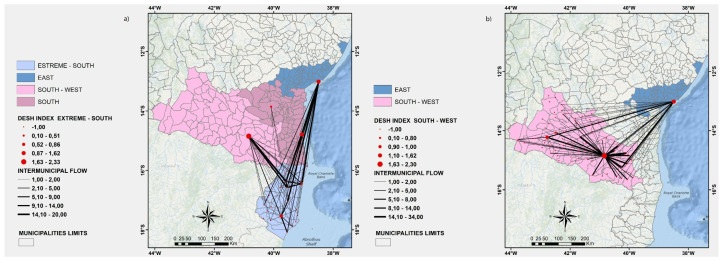
(**a**) A visualization of the Extreme-South, East, South-West, and South regions. (**b**) A visualization of the East and South-West regions. Source: Author.

**Figure 5:**
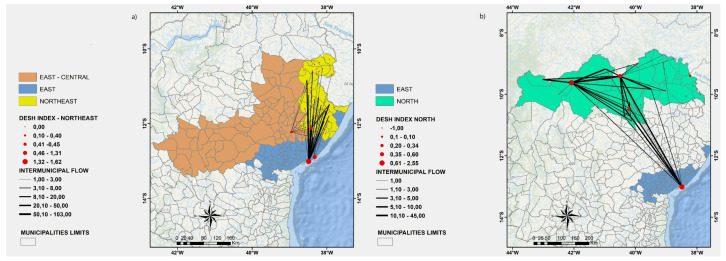
(**a**) A visualization of the East-Central, East, Northeast regions. (**b**) A visualization of the East and North regions. Source: Author.

**Figure 6:**
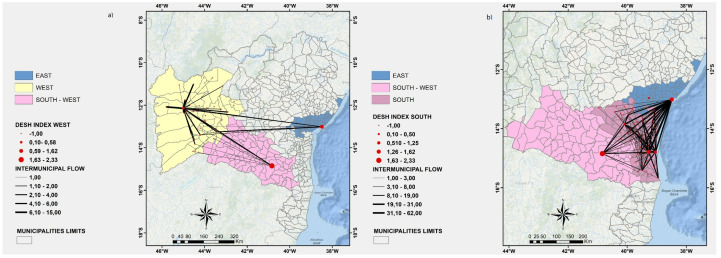
(**a**) A visualization of the East, West, South-West regions. (**b**) A visualization of the East, South-West and South regions. Source: Author.

In Figure 3, Figure 4, Figure 5a, and Figure 6, we show a more detailed visualization of the observed hospitalization networks previously shown in Figure 1b. These figures detail the transportation network for hospitalized COVID-19 patients with a focus on the relation between distinct regions of the state of Bahia and the East region, which contains the capital of the state of Bahia and the highest number of intensive and non-intensive care units, and concentrates the highest number of imported patients from other regions.

Many patients of the regions that are neighbours of the East region, such as the East-Central (Figure 3a), Northeast (Figure 5a), and South (Figure 6b) regions, are hospitalized in the East region, which may indicate that there is an unbalanced distribution of health care units in those neighbouring regions. Moreover, Figure 3, Figure 4, Figure 5a, and Figure 6 show that the regions that are more distant to the East one, such as the North-Central (Figure 3b) and Extreme-South (Figure 4a) regions, also contribute to the exportation of patients to the East region. On the other hand, both the West (Figure 6a) and North (Figure 5b) regions are able to handle the demand for the hospitalization of their patients, even through the West region exported some patients for hospitalization to the East and Southwest regions, and the North region also exported some cases for hospitalization to the East region.

It is known that the state of Bahia has a health care regulation system, and it can be seen from the results of this research that the logistics included in the process of control and the distribution of care needs to be reviewed. This is not only based on the number of inhabitants per square meter in a health region or city, but also on the need for certain medical specialties and the growth in demand for care, suggesting that this may be the reality in other countries with limited resources.

## 5. Conclusions

As we have shown in this paper, the distribution of intensive and non-intensive care units in the state of Bahia, Brazil, is limited, since many patients had to travel more than 300 km to be hospitalized, as shown in Figure 2. Hence, a redistribution of the available health care units, or alternatively a selective, adaptive expansion of the health care infrastructure in the regions that are exporting most of their patients to be hospitalized in other regions, may contribute to a more successful reduction in the length of these travels.

COVID-19 is a dangerous infectious disease that requires new policies of the governments in order to effectively combat the further prolongation of this pandemic. Such policies could be based on scientific research that considers the local realities of the population and other variables that can provide optimized health facility distribution arrangements. In this sense, the provision and distribution of new hospitals and health care units based on scientific criteria that are capable of caring for and admitting patients with COVID-19 would allow for the faster and more effective treatment of these patients.

Finally, we hope that the publication of this manuscript will be an encouraging factor for the state government of Bahia, and other governments around the world, to reproduce the methodology presented in this paper, so as to better evaluate their care unit distribution policies. This approach allows for a clear visualization of demand pressure and migration between different regions, which can help to determine whether the expected strategies previously planned are being observed in practice and thus reducing the social and economic impact and, most importantly, saving lives.

## Figures and Tables

**Table 1:** A numerical overview of the observed hospitalization networks for COVID-19 patients with respect to the nine regions of the state of Bahia.

Region	Patients That Live in That Region	Patients Imported from Other Regions	Patients Exported to Other Regions
North	158 (81.9%)	0 (0.0%)	35 (18.1%)
Northeast	11 (3.7%)	0 (0.0%)	290 (96.3%)
North-Central	12 (12.9%)	2 (2.2%)	79 (84.9%)
East-Central	77 (10.7%)	70 (9.7%)	571 (79.5%)
East	1686 (54.9%)	1369 (44.6%)	14 (0.5%)
West	62 (67.4%)	0 (0.0%)	30 (32.6%)
Southwest	263 (54.3%)	140 (28.9%)	81 (16.7%)
South	401 (47.3%)	61 (7.2%)	385 (45.5%)
Extreme-South	74 (31.8%)	1 (0.4%)	158 (67.8%)

## Data Availability

The original database used is public and available at https://bi.saude.ba.gov.br/transparencia/ (accessed date: 22 October 2021), https://opendatasus.saude.gov.br/dataset/srag-2020 (accessed date: 22 October 2021), and https://opendatasus.saude.gov.br/dataset/srag-2021-e-2022 (accessed date: 22 October 2021). Summarized data from hospitalizations used in the analysis can be found at https://github.com/dataNPAI/UTIdata.git (accessed date: 5 January 2022).

## Abbreviations

The following abbreviations are used in this manuscript:

CNPq	National Council for Scientific and Technological Development
COVID-19	Coronavirus Disease 2019
DESH	Degree of External Search for Hospitalization
FAPESB	The Foundation for Research Support of the State of Bahia
HDI	Human Development Index
SESAB	Health Secretary of the State of Bahia
SUS	Brazilian National Health System
WHO	World Health Organization
